# Survival and Behavior of Encapsulated Probiotics (*Lactobacillus plantarum*) in Calcium-Alginate-Soy Protein Isolate-Based Hydrogel Beads in Different Processing Conditions (pH and Temperature) and in Pasteurized Mango Juice

**DOI:** 10.1155/2019/9768152

**Published:** 2019-02-13

**Authors:** Ong-Ard Praepanitchai, Athapol Noomhorm, Anil Kumar Anal

**Affiliations:** Department of Food Agriculture and Bioresources, Asian Institute of Technology, Pathum Thani, 12120, Thailand

## Abstract

Hybrid alginate-soy protein isolate-based hydrogel beads were prepared and evaluated to enhance the survival of the encapsulated probiotics (*Lactobacillus plantarum*) during heat processing to incorporate in mango juice. The solutions of sodium alginate-soy protein isolate (SA-SPI) with probiotic cells were dropped into the gelation bath containing calcium chloride (3% w/v) solution to develop various types of hydrogel beads. The level of survival of probiotics in encapsulated beads under acidic conditions (pH 2, 3, and 6.5) and bile salt (0.5 and 1.0% w/v) was evaluated. The survival of the encapsulated probiotics to thermal processing was evaluated by treating the beads in saline solution (0.9% w/v) at 30, 50, 63, and 72°C. The encapsulated probiotic bacteria were found alive even after treatment at 72°C for 90 s. Most of the free cells did not survive at the temperature higher than 50°C and very low pH (pH 2 and 3). The survival of probiotic cells was found higher with the hybrid hydrogel beads containing alginate and soy protein isolate (1:8 w/w). Furthermore, mango juice fortified with encapsulated* L. plantarum* in hydrogel beads was subjected to thermal pasteurization at 72°C for 90 s.

## 1. Introduction

The functional beverage business has recently launched the new ready-to-drink with enhanced bioactive compounds. The functionality of food and beverage is enhanced via biological conversions such as by exploiting the activity of probiotics such as lactic acid bacteria (LAB) with a long history of safe use in the food industry. Probiotics have commonly been added to a wide range of food and beverage products such as yoghurt, sour milk, fermented vegetables, and fruit juices [1–3]. Probiotics have numerous health benefits to the human such as improving intestinal microbial balance, inhibiting pathogenic growth by producing antimicrobial substances, simulating and modulating the innate immune systems, exhibiting antimutagenic activities, and preventing carcinogenesis [4, 5]. The genera* Lactobacillus* and* Bifidobacterium *are the most important probiotic microorganisms commonly associated with gastrointestinal tract [2]. The probiotics to be used in food and beverage products should optimally fulfill all of the following criteria: (1) cells remaining viable during industrial processes; (2) survival during preparation and storage of the carrier foods; (3) survival in the gastrointestinal environment of the host; and (4) having ability to provide health benefits through fermentation process in lower intestine of the host [6]. However, most of the probiotics incorporated in food and beverage are sensitive to processing and environmental factors including low pH and heat [7].

Encapsulation of probiotics improves the stabilizing viability during processing and gastrointestinal transit (GIT). Alginate is one of the most frequently used biopolymers for encapsulating the cells and bioactive compounds because of its simplicity, nontoxicity, biodegradability, biocompatibility, low cost, and especially its pH-sensitive profile [1, 8]. The survival of encapsulated* Bifidobacterium bifidum* RO71 in polysaccharide-protein gel beads decreased by about 2 log CFU mL^−1^ after treatment with simulated gastric fluid (pH 2.5) for 2 h. Most of the free cells did not survive at the same condition. Microencapsulated* Lactobacillus casei* NCDC 298 in alginate beads showed a higher survival rate than that of the free probiotics at low pH (1.5) and high bile salt concentration and under heat treatment (55, 60, and 65°C) [9]. The combination of denatured whey protein isolate (WPI) and sodium alginate provided better protection under simulated gastric conditions as well as under pasteurization temperatures [10]. Viable cell numbers of nonencapsulated* L. acidophilus* readily decreased in harsh environment conditions, such as gastric (low pH in stomach) and thermal conditions (higher temperatures during pasteurization), when compared with hydrogel beads comprising WPI and alginate [7]. Similarly, the encapsulated probiotic bacteria with alginate and fish gelatin protein were found viable during exposure at higher temperature (50°C for an hour) [11].* Lactobacillus reuteri* encapsulated in chitosan-alginate beads was found to have better tolerance toward stress conditions encountered in food processing as well as to preserve its functional properties [12].

None of the studies has yet been reported about alginate-soy protein isolate (SPI) based hydrogel beads incorporating probiotic cells. Soy protein isolate (SPI) possesses nonpolar and polar functionalities with both acidic- and basic-charged amino acids that are suitable for encapsulating various active substances. This study aims to determine the synergetic effects from the combination of alginate and SPI as encapsulation matrices, using the widely employed extrusion gelation technique, on the survival of* L. plantarum* probiotics under acidic conditions, and improve the survival of probiotic bacteria in fruit juices during pasteurization.

## 2. Experimental Study

### 2.1. Materials

Alginic acid (sodium salt) extracted from brown algae (molecular weight 200 kD) with a glucuronic acid (60%) and mannuronic acid (40%) were obtained from Rama Production Co. Ltd. (Bangkok, Thailand). Soy protein isolate (SPI) was obtained from Shandong Sinoglory Health Food Co., Ltd. (Qingdao, China). The viscosity of the solutions with different ratios of biopolymers (alginate and soy protein isolate) was measured by Brookfield viscometer (The DV-II+ PRO) with a spindle LV-3 at 25°C. The test was performed three times for each sample.

Pure culture of* L. plantarum* (TISTR 050) was acquired from Thailand Institute of Scientific and Technological Research (TISTR), Thailand. Mahachanok mango puree was purchased from SWIFT Co. Ltd. (Nakornpathaom, Thailand). Culture media and all other analytical grade chemicals were of purchased from Merck (Darmstadt, Germany).

### 2.2. Preparation of L. plantarum Probiotics Culture

The* L. plantarum* TISTR 050 was cultured in MRS medium at 37°C. The cells in early stationary phase were harvested by centrifugation at 5000 rpm (Centrikon T-324, Kontron Instrument, Germany) for 10 min at 4°C. The cell pellets were further washed twice with 10 mL of saline solution (0.9% w/v) followed by centrifugation at 5000 rpm (Centrikon T-324, Kontron Instrument, Germany) for 10 min at 4°C.

### 2.3. Preparation of Hydrogel Bead with L. plantarum

**Figure 1:**
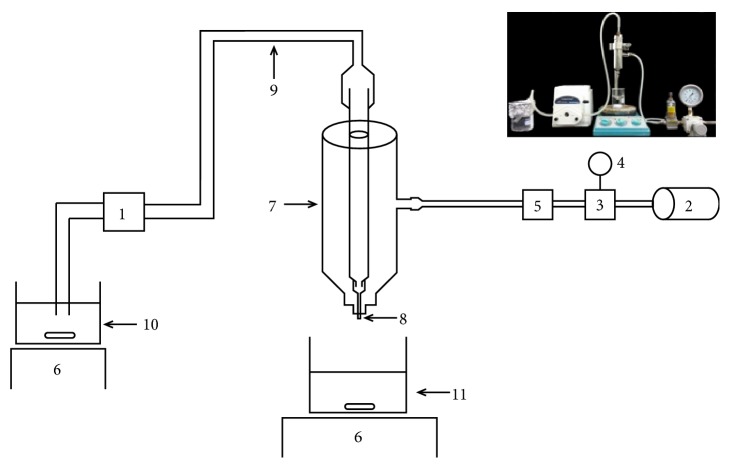
Schematic diagram and working encapsulator (designed and fabricated of low cost encapsulation machine): peristaltic pump (1); air pump (2); pressure regulator (3); pressure gauge (4); air filter membrane (5); magnetic stirrer control (6); double stainless steel jacket (7); needle (8); rubber tube (9); mixture solution (10); gelling beaker (11).

Various types of hydrogel beads were prepared and named after their multivalent components: alginate, soy protein isolate. Alginate-soy protein isolate-based hydrogel beads were produced by the gelation method as described by Bhopatkar et al. [13] with slight modifications. The stock solutions of 4% (w/v) sodium alginate and 20% (w/v) of soy protein isolate proteins in distilled water were prepared. Various ratios of homogenous aqueous solutions of alginate and soy protein isolate (as shown in Table 1) were used as wall materials to encapsulate probiotic cells. The mixtures were then mixed with the suspension comprising* L. plantarum* TISTR 050 probiotics (10^11^ CFU mL^−1^). The encapsulated beads were produced by extrusion technique using encapsulator as shown in Figure 1. The modified encapsulator was designed and fabricated at the Bioprocess Technology Laboratory at the Asian Institute of Technology (AIT), Thailand. The different mixture solutions comprising alginate, soy protein isolates, and probiotic cells (Table 1) were dropped into the gelation bath containing calcium chloride solution (3% w/v) using a hypodermic needle (21 gauge) at a constant flow rate of 3.6 mL min^−1^ using a peristaltic pump (Millipore, USA). After 30 min of incubation for gelation, the hydrogel beads were washed twice with saline solution (0.9% w/v) and further stored at 4°C for further use.

### 2.4. Particle Size Determination of Hydrogel Beads

The particle size of the 100 hydrogel beads was measured with a micrometer (Mittotuyo micrometer, NSK Co. Ltd., Japan). The average values are presented.

### 2.5. Encapsulation Efficiency

The encapsulation efficiency of the probiotic cells in alginate-soy protein isolate hydrogel beads was determined by digestion method as described by Anal and Stevens [1] with slight modification. The probiotic cells loaded hydrogel beads (1 g) were transferred in 9 mL of phosphate buffer saline solution (0.1 M PBS; pH 7.4) at 4C for 24 h suspension was then centrifuged at 6000 rpm (Centrikon T-324, Kontron Instrument, Germany) for 30 min. The cell pellets were plate-counted and the encapsulation efficiency of probiotic cells (CFU) was determined as follows:

(1) $$\begin{array}{c} Encapsulation\;\;efficiency\left(\;\%\;\right)\text{=}\frac{Cells\;\;count\;\;\left(CFU/mL\right)\;\;after\;\;disintegration\;\;of\;\;the\;\;hydrogel\;\;beads}{Initial\;\;loading\;\;of\;\;the\;\;cells\;\;\left(CFU/mL\right)\;\;in\;\;hydrogel\;\;beads}\times 100 \end{array}$$

### 2.6. Surface Morphology of Hydrogel Beads

The shape and surface morphology of hydrogel beads loaded with* L. plantarum *were observed under scanning electron microscopy (SEM, Hitachi S-3400N, Japan). The hydrogel beads were mounted on copper stubs followed by sputter-coated in a metallizer (Agar Sputter Coater) with gold-palladium to reach a thickness of coating of 100 A° and then observed in high vacuum mode in different magnifications.

### 2.7. Evaluation of Survival of Encapsulated Probiotics in Acidic Solutions

The survival of the* L. plantarum* probiotics encapsulated in the hydrogel beads under acidic conditions was performed at pH 2.0, 3.0, and 6.5. Free cells were used as control.* L. plantarum-*encapsulated in various hydrogel beads (10^10^ CFU mL^−1^) were added to test tubes containing 9 mL of NGYC medium (comprising nonfat skim milk (12% w/v), glucose (2% w/v), yeast extract (1% w/v), and cysteine (0.05% w/v) and adjusted to the desired pH with 5 M HCl or 1 M NaOH). After incubation at 37°C for 3 h, the samples were centrifuged (5000 rpm for 10 min at 4°C) and the hydrogel beads were further allowed to disintegrate in 9 mL phosphate buffer saline solution (0.1 M; pH 7.0) to release the cells from the beads. The survival of the free and encapsulated* L. plantarum* probiotics was evaluated by standard plate count (SPC) method.

### 2.8. Evaluation of Survival of Encapsulated Probiotics in Different Bile Salt Solutions

Free cells and encapsulated* L. plantarum *were evaluated for their survival in 0, 0.5, and 1.0% (w/v) porcine bile salt solutions following the method of Hansen et al. [14] with slight modifications. Briefly, free cell and* L. plantarum-*encapsulated beads (10^10^ CFU mL^−1^) were added to different test tubes containing 9 mL of milk–yeast extract medium at pH 6.9 (10% w/v nonfat skim milk, 0.5% w/v yeast extract, 0.05% w/v cysteine) containing 0.5, 1.0% (w/v) porcine bile salt. The samples were further incubated at 37°C for 3 and 6 h. The viable cell counts were enumerated as described in earlier section.

### 2.9. Evaluation of Survival of Encapsulated Probiotics under Heat Treatment

The viability of* L. plantarum-*encapsulated hydrogel beads dispersed in normal saline solution (0.9% w/v) was exposed to heat treatment following the method descried by Fang et al. [7] with slight modifications. Free cells and* L. plantarum-*encapsulated beads (10^10^ CFU mL^−1^) were placed in test tubes containing 9 mL of normal saline solution (0.9% w/v). The test tubes were further incubated in a water bath at various temperatures (30, 50, 63, and 72°C) for 2 min. Aliquots were collected at different intervals of time after incubation. The samples were cooled down to room temperature (~25°C). The viability of the free and encapsulated* L. plantarum* probiotics was obtained as described above for heat treatment using SPC.

### 2.10. Evaluation of Survival of Encapsulated Probiotics in Mango Juice during Pasteurization

The mango juice (50% w/v) was prepared by dissolving the mango concentrate in sterilized deionized water. Free cell and* L. plantarum *were placed in test tubes containing 9 mL of preheated mango juice. The test tubes were incubated in a water bath at 72°C for 90 s followed by storing at 4°C. The samples were further taken every 7 days to analyze the probiotic cells* in juice* and pH. All experiments were conducted in triplicate.

### 2.11. Analysis of Titratable Acidity

The pH of mango juice was measured using pH meter (Hanna pH 211, USA). Titratable acidity (TA) was determined using titration method with standardized alkali solution (0.1 N NaOH). TA expressed as the percentage of citric acid per 100 g of juice.

### 2.12. Statistical Analysis

All experiments were repeated at least three times. Results are reported as mean ± standard deviation using IBM SPSS statistics program (Ver. 21, USA). The statistical significance among various samples of free cell and encapsulated beads was evaluated with one-way ANOVA, with significant level (*p* < 0.05).

## 3. Results and Discussion 

### 3.1. Formation of Encapsulated Probiotics SA:SPI Hybrid Hydrogel Beads

**Figure 2:**
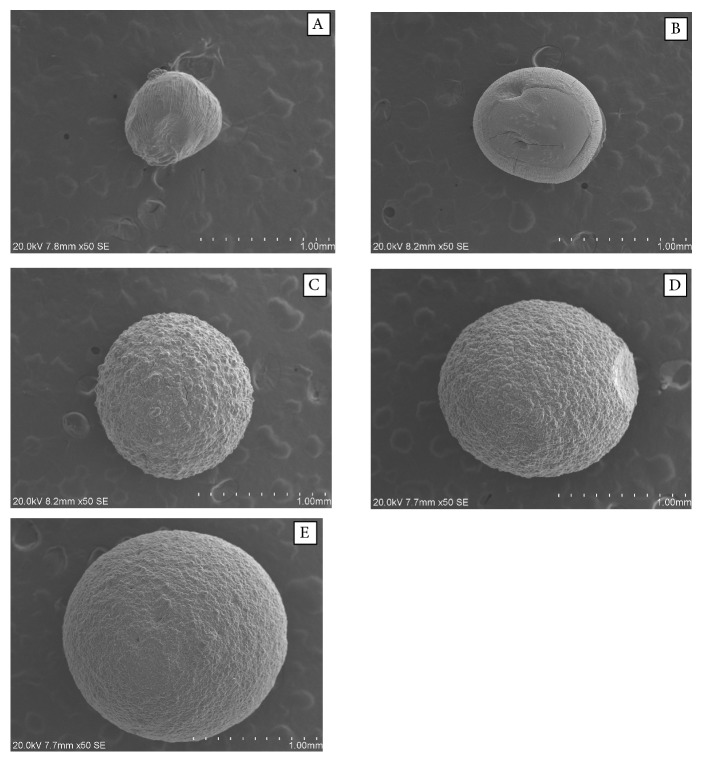
*Scanning Electron Microscopy* (SEM) images (magnification 50×) showing the increased trends of sodium alginate (SA)—soy protein isolate (SPI) hydrogel beads containing* Lactobacillus plantarum *with the increasing SA: SPI ratios (a) bead A (1 : 0), (b) bead B (1 : 2), (c) bead C (1 : 4), (d) bead D (1 : 8), and (e) bead E (1 : 12).

**Figure 3:**
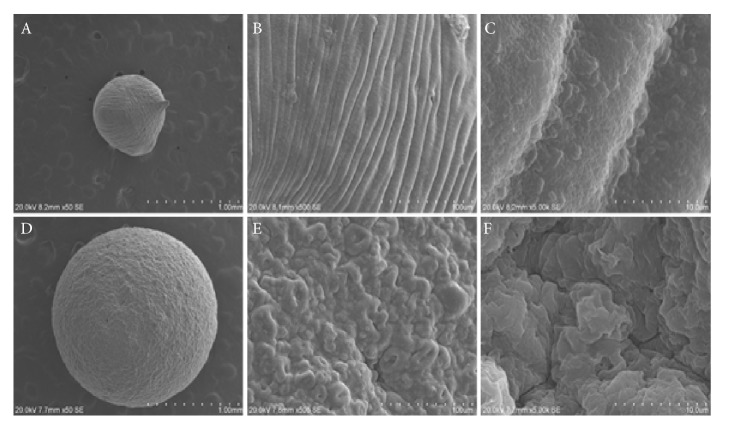
*Scanning Electron Microscopy* (SEM) images (magnification 50×) showing the surface morphology of sodium alginate (SA)—soy protein isolate (SPI) hydrogel beads containing* Lactobacillus plantarum. *(A) Only sodium alginate hydrogel bead (magnification 50X); (B) only sodium hydrogel bead (magnification 500X), (C) only sodium hydrogel bead (magnification 5000X), and (D) sodium alginate-soy protein isolate hydrogel beads (ratio 1:8; magnification 50X); (E) sodium alginate-soy protein isolate hydrogel beads (ratio 1:8; magnification 500X); sodium alginate-soy protein isolate hydrogel beads (ratio 1:8; magnification 5000X).

The polyanionic polymer solution sodium alginate in aqueous solution dropped into an aqueous solution containing a suitable divalent counter cation like Ca^2+^ has the tendency to form hydrogel beads using ionotropic gelation method. The probiotic cells were encapsulated in calcium-alginate-SPI based hydrogel beads. Table 1 shows the formulation of different ratio of SA and SPI (A-E formulations), their viscosity, size of the hydrogel beads, and encapsulation efficiency of probiotic cells. The size of the hydrogel beads was found increasing with the increased viscosity of the hydrogel mixtures as shown in Table 1 and illustrated in Figure 2. The encapsulation efficiency of probiotic cells was found 90-92% in all types of hydrogel beads.  Figure 3 illustrates the surface morphology of the hydrogel beads encapsulating probiotic cells. The smooth and spherical hydrogel beads were formed dropping the alginate alone and the mixtures of alginate with various concentrations of soy protein isolate (SPI). The gelation of the two biopolymeric mixtures (sodium alginate and SPI) is likely to occur via electrostatic complexion and the Ca^2+^ ions neutralize electrostatic repulsion and form salt bridges between the biopolymeric networks [15, 16]. The structure of outer wall provides an efficient protective barrier to diffusion toward the core of the capsule. As illustrated in Figure 3, the entrapped rod-shaped probiotic cells are clearly observed inside the membranes of the hydrogel beads.

### 3.2. Viability of Encapsulated L. plantarum under Acid Solutions

**Figure 4:**
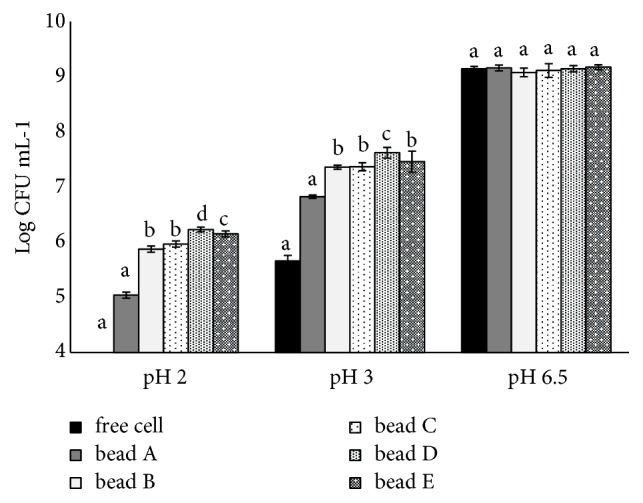
Survival of free cell and encapsulated probiotics in SA beads (bead A formulation) and SA/SPI beads (bead B to E formulations) under acidic conditions at 37°C for 3 h.

Figure 4 illustrates the viability of probiotic cells encapsulated in SA and SA/SPI beads in acidic solutions. The viability of both the free and the encapsulated probiotics (in SA and SA/SPI beads) increased with decreased acidity from pH 2 to 6.5. At pH 6.5; most of the free and encapsulated probiotics were found viable and active after 3 h of incubation. In contrast, at pH 2 and 3, the survival of the encapsulated probiotics was significantly (p > 0.05) higher than that of the free probiotics owing to their protection from direct contact with the acidic medium as a result of encapsulation. The free cells did not survive at pH 2 after 3 h of incubation. The viable cell count of encapsulated bead in the SA/SPI beads (bead A-E formulations) at pH 2 was 5.04 ± 0.05, 5.88 ± 0.06, 5.97 ± 0.06, 6.23 ± 0.04, and 6.15 ± 0.05 log CFU mL^−1^, respectively. More importantly, the survival of the probiotics encapsulated in the SA/SPI beads was higher than that of the probiotics encapsulated in SA beads only. Soybean protein isolate (SPI) seems to exert a synergetic effect on the survival of encapsulated probiotics. The results additionally indicated that, at pH 2 and 3, increasing the content of SPI at a given SA content (i.e., SA:SPI ratios from 1:2 to 1:12% w/w) influenced the survival of probiotic cells. The probiotic in bead D formulation achieved the highest survival rate (p < 0.05) with 6.23 ± 0.04 and 7.62 ± 0.10 log CFU mL^−1^ when submerged in NGYC medium pH 2 and 3 for 3 h, respectively. The variations in the probiotics survival may be due to differing complexion between SPI and SA when the relative content of SPI exceeds 8% w/v (at 1% SA).

### 3.3. Viability of Encapsulated L. plantarum under Different Bile Salt Solutions

Survival of free and encapsulated probiotics in SA and SA/SPI hydrogel beads in different bile salt concentrations (0.5 and 1.0 %, w/v of porcine bile salt solutions; pH 6.9) at 37°C was also evaluated. Viability of free and encapsulated probiotics in SA hydrogel bead remained unchanged after 6 h of incubation in milk–yeast extract medium without bile salt. In contrast, the viability of the encapsulated probiotics in the SA/SPI beads increased gradually from 3 to 6 h of incubation with increasing SA/SPI ratios to 1:8. This is due to the growth of the probiotics encapsulated in the SA/SPI beads; a milk–yeast extract medium, used as nutrient, penetrated the interface between the electrostatically charged SA and SPI complexion particles and the SA wavy matrix. Increasing the bile salt concentrations from 0.5 to 1.0 % (w/v), the survival of the free cells and the probiotic cells encapsulated in SA beads only decreased with increasing incubation periods from 3 to 6 h. The viability of free cells and encapsulated probiotic in SA beads after 6 h of incubation in 0.5% w/v bile salt was 8.17 ± 0.04 and 8.62 ± 0.08 log CFU mL^−1^, respectively, and 1.0% w/v bile salt of 6 h incubation was 8.05 ± 0.20 and 8.51 ± 0.10 log CFU mL^−1^, respectively. However, the survival of the probiotic in the SA/SPI beads remained nearly the same as that of the starting content (9 log CFU mL^−1^) after 6 h owing to protection from the electrostatically charged SA and SPI complexion in the hybrid beads. Hence, bile salt concentrations of 0.5 and 1.0% w/v (high bile) had no diminishing effects on the survival level of the probiotics encapsulated in the SA/SPI beads after 6 h of incubation.

### 3.4. Viability of L. plantarum-Encapsulated in SA/SPI Beads under Heat Treatment

**Figure 5:**
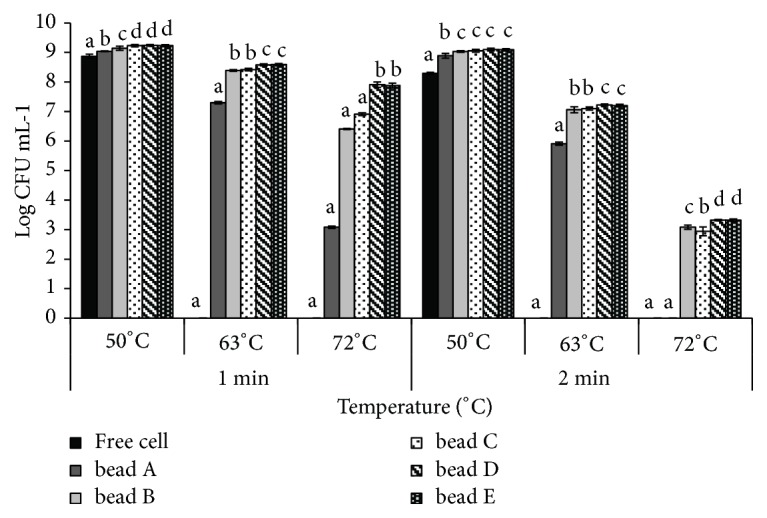
Survival of free and encapsulated probiotics in SA beads (bead A formulation) and SA/SPI beads (bead B to E formulations) under heat treatment at 50°C, 63°C, and 72°C for 1 min and 2 min.

In order for probiotic cells to be effective and remain viable in food and beverage products, they must withstand the recommended pasteurization temperatures and/or other industrial processing parameters. Survival of probiotic cells in SA/SPI beads was therefore evaluated under the heat treatment at 50, 63, and 72°C and illustrated in Figure 5. All the free cells were killed at within a min. In contrast, addition of SPI in beads significantly improved the survival of the probiotic cells in SA/SPI beads. Increasing content of SPI at a given SA content (i.e., SA:SPI ratios from 1:2 to 1:12% w/w) also enhanced the survival of the probiotic cells; however, the probiotic in the SA/SPI beads prepared at a SA/SPI ratio of 1:8% w/w achieved the highest survival rate (7.91 ± 0.09 log CFU mL^−1^) after heat treatment at 72°C for 1 min. There was no significant difference (p < 0.05) between the survival of probiotic in beads D and E formulations. However, the survival of probiotic in SA beads was not found after treat with heat at 72°C for 2 min. Importantly, adequate active amount of probiotic (6-8 log CFU mL^−1^) in the bead B to E formulations survived for 1 min under heat treatment at 63 and 72°C. The results confirmed the presence of probiotic bacteria at minimum levels of 6-7 log CFU mL^−1^ which is recommended in functional foods [17–19]. Fang et al. [7] reported that the cells encapsulated with whey protein and alginate beads provided better thermal protection on the viability of* L. acidophilus* more than free and alginate encapsulated cells.

### 3.5. Viability of L. plantarum-Encapsulated SA/SPI Beads in Mango Juice after Pasteurization

**Figure 6:**
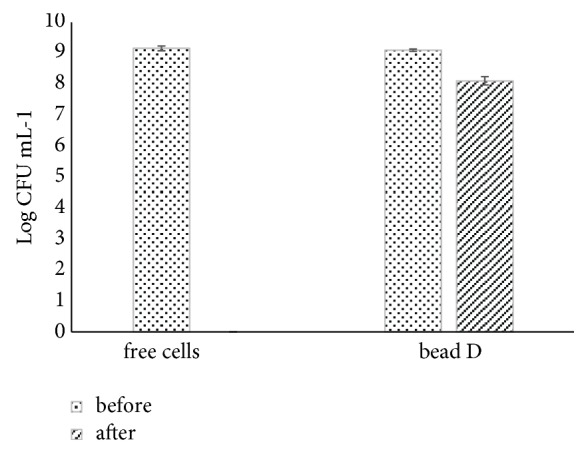
Survival level of the probiotics free cells and encapsulated in SA/SPI beads (bead D formulation) before and after pasteurization process.

The hydrogel beads (formulation D) were chosen to fortify with mango juice.  Figure 6 illustrates the survival of free and encapsulated cells in mango juice after pasteurization process at 72°C for 90 s. The free cells were found killed in mango juice after exposure to pasteurization temperature (72°C, 90 s). In case of mango juice with the cells encapsulated in bead D formulation, only one Log CFU mL^−1^ (from 9.10 ± 0.04 log CFU mL^−1^ to 8.11 ± 0.13 log CFU mL^−1^) was found decreased. Therefore, it was proven that the encapsulated probiotic bacteria with SA/SPI can protect probiotics from thermal pasteurization.

### 3.6. Viability of L. plantarum TISTR 050 in Mango Juice during Storage

**Figure 7:**
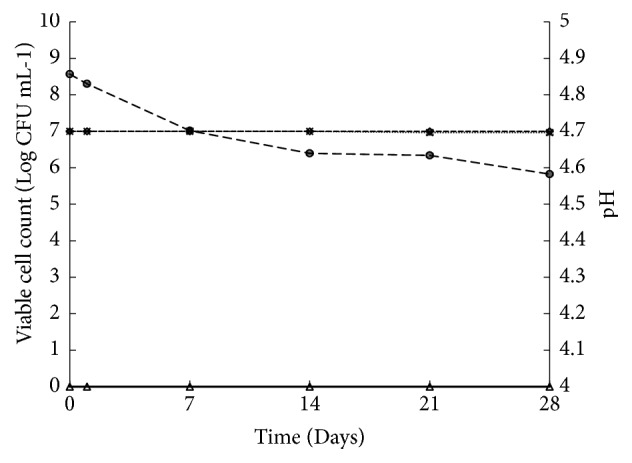
The survival of* L. plantarum* TISTR 050 and pH change in mango juice after pasteurization during storage at 4°C for 35 days; survival of free cell (○), survival of encapsulated bead (∆), pH of free cell (*◇*), and pH of encapsulated bead (×).

Figure 7 illustrates the changes in numbers of viable probiotic cells in in mango juice during storage at 4°C over 28 days. A drop in the microbial population of pasteurization mango juice with encapsulated* L. plantarum* bead was observed during the storage. The survival of* L. plantarum* TISTR 050 beads in mango juice was decreased from 8.56 ± 0.02 log CFU mL^−1^ to 5.83 ± 0.03 log CFU mL^−1^ during 28 days of storage. The number of cell during storages is decreased with large amount when compared to another study [20], due to* L. plantarum* in encapsulated bead of our study passing the pasteurization process and this is the main cause of making the cell injury and decreasing to almost 2.8 log CFU mL^−1^ during storage. However, there was not any microorganism on pasteurized mango juice with free cell all the period of storage found. Therefore, encapsulated probiotics in SA/SPI bead were able to maintain viability in acidic fruit juice up to 25 days after pasteurization in which the probiotic remained at least 6 log CFU mL^−1^.

### 3.7. pH and Acidity Changes during Mango Juice Storage

The initial pH of all mango juice samples at day 0 (before the addition of* L. plantarum* TISTR 050) was 4.7. The pH of pasteurized mango juice with free cell remained constant during storage (28 days) because all* L. plantarum* was killed during pasteurization as illustrated in Figure 7. The pasteurized mango juice with encapsulated* L. plantarum* bead remained constant at 4.7 during storage. In case of acidity of both pasteurized mango juice with free cell and pasteurized mango juice with encapsulated* L. plantarum* TISTR 050, the hydrogel beads were constant during 35 days of storage (1.25%). There was no significant difference (*ρ* < 0.05) between the pH and the acidity at day 0 and day 28 for two types of stored juice. Therefore, it was found that encapsulation of probiotic was able to maintain the pH and acidity of fruit juice during storage. As illustrated in Figure 7, for the acidity of both pasteurized mango juice with* L. plantarum* TISTR 050 free cells and pasteurized mango juice with encapsulated* L. plantarum* TISTR 050, the hydrogel beads remained constant during 35 days of storage (1.25%). There was no significant difference (p < 0.05) of acidity at day 35 between two types of storage juices.

## 4. Conclusions

Encapsulated beads of* L. plantarum* (TISTR 050) probiotics were successfully obtained by extrusion gelation technique of various ratios of pure SA and SA/SPI into CaCl_2_ solution. The encapsulated beads with SA and SA/SPI can protect* L. plantarum* from gastric condition; however, SA/SPI beads provided better protection. The bile salt concentrations of 0.5 and 1.0% (w/v) had no diminishing effects on the survival level of the probiotics encapsulated in the SA/SPI beads after 6 h of incubation. Furthermore, the probiotics encapsulated in the SA/SPI beads survived longer relative to those encapsulated in the SA beads and the nonencapsulated probiotics subjected to heat treatment. The application of encapsulated bead of* L. plantarum* in mango juice under pasteurization process showed successful resistance to thermal conditions (72°C for 90 s). The similar result is reported in case of encapsulated probiotics in alginate—whey protein isolate-based hydrogel beads [7]. The pH of mango juice with encapsulated bead remained constant at 4.70 during storage. Probiotics were found heat resistant during pasteurization process and thus found in higher concentration in mango juice containing the probiotics loaded SA/SPI beads; SPI is an alternative encapsulated material to create a barrier to the surrounding environment and it is suitable for the people who are allergic to dairy proteins.

## Figures and Tables

**Table 1:** Different ratio of biopolymers, their viscosity, and effects on hydrogel bead size and encapsulation efficiency.

Formulations	SA: SPI (% w/w)	Viscosity (cp)	Bead size (*μ*m)	Encapsulation Efficiency (%)
A	1:0	164.67 ± 4.03^a^	3030 ± 30^a^	90.60 ± 0.90^a^
B	1:2	250.17 ± 7.56^b^	3090 ± 40^b^	92.00 ± 1.00^ab^
C	1:4	423.53 ± 10.08^c^	3160 ± 40^c^	92.70 ±1.00^b^
D	1:8	1040.53 ± 49.75^d^	3320 ± 40^d^	91.60 ± 1.00^b^
E	1:12	2580.67 ± 53.78^e^	3440 ± 60^e^	91.50 ± 1.30^ab^

SA: sodium alginate; SPI: soy protein isolate. Means followed by the same letter in each column do not differ by least significant difference (LSD) at *p* < 0.05. Data are means ± standard deviation of three replications.

## Data Availability

All relevant data is included within manuscript.
